# Kawasaki Disease-Like Features in 10 Pediatric COVID-19 Cases: A Retrospective Study

**DOI:** 10.7759/cureus.11035

**Published:** 2020-10-19

**Authors:** Noor Ul Falah, Shahnawaz Hashmi, Zahoor Ahmed, Ali Jaan, Ali Akhtar, Farhan Khalid, Umar Farooque, Muhammad Tayyab Shera, Sundas Ali, Ayesha Javed

**Affiliations:** 1 Internal Medicine, Mayo Hospital, King Edward Medical University, Lahore, PAK; 2 Internal Medicine, Army Medical College, National University of Medical Sciences, Rawalpindi, PAK; 3 Neurology, Dow University of Health Sciences, Karachi, PAK; 4 Internal Medicine, Holy Family Hospital, Rawalpindi Medical University, Rawalpindi, PAK

**Keywords:** coronavirus disease, covid-19, children, sars-cov-2, coronavirus, respiratory tract infection, pediatric, kawasaki disease, inflammatory illness

## Abstract

Children with coronavirus disease 2019 (COVID-19), which is caused by severe acute respiratory syndrome coronavirus type 2 (SARS-CoV-2), usually present with symptoms of mild upper respiratory tract infection without developing any significant complications. However, this observation has been rendered incautious by hundreds of clinical cases from around the world that have depicted a less benign multisystem inflammatory illness mimicking Kawasaki disease in COVID-positive pediatric patients. Our study aimed at retrospectively reviewing the different features of Kawasaki disease-like illness in children suffering from COVID-19, including the complications, laboratory investigations, treatment strategies used during their hospital stay, and outcomes. We searched the electronic database of the two pediatric units of Mayo Hospital, Lahore, Pakistan, for children who had been admitted to the ward between April 2020 and July 2020 and were diagnosed with COVID-19. A total of 10 such pediatric cases were found, whose clinical details were then reviewed and the obtained data were presented in the form of tables and percentages. The median age was between 4 months to 11 years (mean: 6 years). Of the 10 patients, 8 (80%) were boys. Criteria for Kawasaki disease were met in all of them (100%), with a complete presentation in five (50%). Fever (100%), conjunctival and oral cavity changes (90%), and rash (80%) were the most common features. Seven (70%) patients required admission to a critical care unit, but no mortality occurred. This article can assist in understanding and dealing with Kawasaki disease-like manifestation of pediatric COVID-19 infection, especially in critical care settings, and its possible complications. It will help in a timely and appropriate decision-making regarding treatment and management of such cases.

## Introduction

In 1967, Tomisaku Kawasaki described Kawasaki disease (KD) as a rare systemic vasculitis occurring in children ≥5 years of age. Its etiology is still unknown, but an infectious trigger has been proposed. The diagnostic criteria for KD set by the American Heart Association include the following parameters: ≥5 days of fever, oral mucosal changes, bilateral nonexudative conjunctivitis, exanthematous rash, desquamation of hands and feet, and cervical lymphadenopathy. Apart from hyperthermia, at least four of the five major clinical features are required for complete and less than four for an incomplete form of KD [1].

Since its outbreak in December 2019, the severe acute respiratory syndrome coronavirus type 2 (SARS-CoV-2) infection has caused 30,624,590 confirmed cases and 953,903 confirmed deaths globally as of September 20, 2020 [2]. An epidemiological study from China showed that 90% of the 731 laboratory-confirmed COVID-19 patients aged <18 years had asymptomatic, mild, or moderate infection, thus rendering it relatively benign than the adult infection [3]. Children with symptomatic COVID-19 may develop fever, fatigue, cough, sore throat, rhinorrhea, congestion, and shortness of breath, and the critical cases present with gastrointestinal distress, shock, and respiratory failure, deranged coagulation, and renal shutdown [4]. However, there has been an increase in the incidence of KD-like illness among COVID-positive children, either concomitant with or after the resolution of COVID-19 infection [5]. Some cases develop a shock-like systemic inflammatory illness resembling toxic shock syndrome, which is identified as a multisystem inflammatory syndrome, warranting their admission to the critical care unit [6].

## Materials and methods

We searched the electronic medical records (EMR) of the two pediatric units at Mayo Hospital, Lahore, Pakistan. Our search was limited to the pediatric COVID-19 patients admitted between April 2020 and July 2020. SARS-CoV-2 infection was confirmed on either a real-time reverse transcriptase polymerase chain reaction (RT-PCR) or a positive serology. A total of 10 such cases were found, whose clinical details were then thoroughly reviewed. Inclusion criteria involve the children aged 0-16 years diagnosed with COVID-19. We studied the patients' demographics, comorbidities, signs, and symptoms of their COVID-19 infection, including those of KD, complications, laboratory investigations, treatment strategies utilized in the hospital, and outcomes. One of the included cases had negative PCR and serology, but COVID-19 was diagnosed based on his symptoms, a history of COVID-19 contact, and CT results suggestive of SARS-CoV-2 pneumonia. Another PCR-negative case was suspected of having a past asymptomatic COVID-19 infection because of his locality and travel history of his father. The results were then presented as tables and percentages.

## Results

A total of 10 pediatric cases with a median age of 4 months to 11 years (mean: 6 years) were included. Eight of them (80%) were boys.

Criteria for KD were met in all of them (100%), with a complete presentation in five (50%) cases (Table 1). None reported any previous comorbidity. Polymerase chain reaction (PCR) test for SARS-CoV-2 was positive in seven (70%) patients, whereas one patient (10%) showed positive SARS-CoV-2 IgG serology. One case had negative PCR and serology, but COVID-19 was diagnosed on symptoms, history of COVID-19 contact, and lung CT, which revealed ground‐glass opacities and consolidation in the right posterior basal area (<10% of the lung parenchyma), suggestive of COVID‐19 pneumonia. Another PCR-negative case was suspected of having a past asymptomatic COVID-19 infection because of father's locality and travel history.

**Table 1:** Patient characteristics IgG, immunoglobulin G; N/A, not available

Characteristics	Patient 1	Patient 2	Patient 3	Patient 4	Patient 5	Patient 6	Patient 7	Patient 8	Patient 9	Patient 10
Age and gender	5-year-old boy	3-year-old boy	10-year-old boy	11-year-old girl	6-month-old girl	8-year-old boy	4-month-old boy	5-year-old boy	11-year-old boy	6-year-old boy
Criteria met for Kawasaki disease	Yes	Yes	Yes	Yes	Yes	Yes	Yes	Yes	Yes	Yes
SARS-CoV-2 PCR positive	Yes	No	Yes	Yes	Yes	Yes	Yes	Yes	No	Yes
SARS-CoV-2 serology positive	Not done	Not done	Not done	Not done	Not done	Not done	Not done	Not done	IgG positive	Not done
Sick contacts	N/A	Yes	N/A	N/A	N/A	N/A	N/A	N/A	No	Yes
Travel history	N/A	No	N/A	N/A	N/A	N/A	N/A	N/A	Yes	N/A
Any comorbidity	No	No	No	No	No	No	No	No	No	No

The symptoms of KD included fever (n = 10) (100%), conjunctival and oral cavity changes (90%), rash (80%), extremity changes (50%), and cervical lymphadenopathy (30%) (Table 2).

**Table 2:** Features of Kawasaki disease

Kawasaki Feature	Patient 1	Patient 2	Patient 3	Patient 4	Patient 5	Patient 6	Patient 7	Patient 8	Patient 9	Patient 10
Complete presentation (fever >4 days and ≥4 principal criteria)	No	Yes	No	No	Yes	Yes	Yes	No	No	Yes
Fever with duration	Yes, 8 days	Yes, 8 days	Yes, 7 days	Yes, >4 days	Yes, >4 days	Yes, >4 days	Yes, >4 days	Yes, >4 days	Yes, >4 days	Yes, >7 days
Rash	No	Yes	Yes	Yes	Yes	Yes	Yes	No	Yes	Yes
Conjunctival injection	Yes	Yes	Yes	No	Yes	Yes	Yes	Yes	Yes	Yes
Lips and oral cavity changes	Yes	Yes	Yes	Yes	Yes	Yes	Yes	No	Yes	Yes
Cervical Lymphadenopathy	Yes	Yes	No	No	No	No	Yes	No	No	No
Extremity changes (swelling, erythema)	No	No	No	Yes	Yes	Yes	No	Yes	No	Yes

Among other symptoms, gastrointestinal problems were prevalent (70%) followed by neurological (30%) and respiratory dysfunctions (20%). Among the gastrointestinal symptoms, diarrhea and abdominal pain were most common (n = 5) (50%). Three (30%) cases developed perineal, periungal, or facial desquamation (Table 3).

**Table 3:** Symptoms and signs GI, gastrointestinal

Symptoms and Signs	Patient 1	Patient 2	Patient 3	Patient 4	Patient 5	Patient 6	Patient 7	Patient 8	Patient 9	Patient 10
Sore throat	No	No	No	Yes	No	Yes	No	No	No	Yes
Respiratory symptoms	No	No	Yes	No	No	Yes	No	No	No	No
Cough	-	-	Yes	-	-	Yes	-	-	-	-
Dyspnea	-	-	Yes	-	-	Yes	-	-	-	-
Stuffy nose/sneezing/rhinorrhea	-	-	-	-	-	-	-	Yes	Yes	Yes
GI symptoms	Yes	No	Yes	Yes	Yes	No	No	Yes	Yes	Yes
Abdominal pain	Yes	-	No	Yes	No	-	-	Yes	Yes	Yes
Diarrhea	Yes	-	Yes	No	No	-	-	No	No	Yes
Vomiting	No	-	No	No	No	-	-	No	No	No
Decreased appetite	Yes	-	No	Yes	Yes	-	-	No	No	No
Dysuria	Yes	No	No	No	No	No	No	Yes	No	No
Neurological symptoms	No	No	No	No	Yes	No	Yes	No	-	-
Irritability	-	-	-	-	Yes	No	Yes	No	-	-
Anosmia	-	-	-	-	No	No	No	No	-	-
Headache	-	-	-	-	No	No	No	No	-	-
Confusion	-	-	-	--	No	No	No	No	-	-
Meningeal signs	-	-	-	-	No	No	No	Yes	-	-
Drowsiness	-	-	-	-	No	No	No	Yes	-	-
Perineal/periungal/facial desquamation	No	Yes	No	No	No	Yes	No	No	Yes	No

Complications included admission to a critical care unit (70%), hypotension/shock (60%), inotropic support (40%), pericarditis/pericardial effusion (40%), myocarditis (30%), coronary artery dilation (20%), acute kidney injury (10%), paralytic ileus (10%), and coronary aneurysm in none (Table 4). None of them required artificial ventilation, and no mortality was reported in any case except for one whose outcome data was not available.

**Table 4:** Complications N/A, not available

Complication	Patient 1	Patient 2	Patient 3	Patient 4	Patient 5	Patient 6	Patient 7	Patient 8	Patient9	Patient 10
Admission to the critical care unit	Yes	No	Yes	Yes	No	Yes	No	Yes	Yes	Yes
Artificial ventilation	No	No	No	No	No	No	No	No	No	No
Hypotension/shock	Yes	No	Yes	Yes	No	Yes	No	Yes	Yes	No
Inotropic support	No	No	Yes	Yes	No	No	No	Yes	Yes	No
Acute kidney injury	No	No	No	Yes	No	No	No	No	No	No
Coronary artery dilatation	No	No	No	No	No	No	Yes	No	Yes	No
Coronary artery aneurysm	No	No	No	No	No	No	No	No	No	No
Myocarditis	No	No	Yes	Yes	No	No	No	Yes	No	No
Pericardial effusion/pericarditis	Yes	No	Yes	No	No	No	No	No	Yes	No
Paralytic ileus	No	No	No	No	No	No	No	No	No	Yes
Mortality	No	No	N/a	No	No	No	No	No	No	No

All the cases reported elevated C-reactive protein levels, whereas raised levels of ferritin (60%), erythrocyte sedimentation rate (ESR) (50%), procalcitonin (50%), and interleukin-6 (10%) were also noted (Table 5). Other significant findings included leukocytosis (90%) and hypoalbuminemia (70%). Raised levels of fibrinogen (40%), D-dimers (30%), lactate dehydrogenase (LDH) (20%), troponin (40%), and N-terminal pro-B-type natriuretic peptide (NT-proBNP) (40%), as well as anemia (50%), thrombocytopenia (30%), and lymphopenia (30%) were seen. Moreover, six (60%) cases had abnormal chest X-ray findings.

**Table 5:** Hospital Investigations CRP, C-reactive protein; ESR, erythrocyte sedimentation rate; IL-6, interleukin-6; LDH, lactate dehydrogenase; N/A, not available; NT-proBNP, N-terminal pro-B-type natriuretic peptide

Inflammatory Markers	Patient 1	Patient 2	Patient 3	Patient 4	Patient 5	Patient 6	Patient 7	Patient 8	Patient 9	Patient 10
CRP (0-5 mg/L)	Raised (250 mg/L)	raised (195 mg/L)	Raised (280 mg/L)	Raised (>300 mg/L)	Raised (133 mg/L)	Raised (317 mg/L)	Raised (115.6 mg/L)	Raised (120 mg/L)	Raised (189.5 mg/L)	Raised (130 mg/L)
Ferritin (7-140 µg/L)	Raised (1030 µg/L)	N/A	Raised (1089 µg/L)	Raised (1789 µg/L)	N/A	Raised (1496 µg/L)	N/A	Raised (600 µg/L)	N/A	Raised (612 µg/L)
ESR (0-22 mm/hr)	Raised (72 mm/hr)	N/A	Raised (57)	N/A	Raised (118)	Raised (115)	N/A	Raised (70)	N/A	N/A
Procalcitonin (<2 μg/L)	Raised (27 μg/L)	N/A	Raised (28 μg/L)	Raised (16.28 μg/L)	N/A	N/A	N/A	N/A	Raised (14.55 μg/L)	Raised (5.05 μg/L)
IL-6 (<8.5 pg/mL)	N/A	N/A	N/A	Raised (1449 pg/mL)	N/A	N/A	N/A	N/A	N/A	N/A
Coagulation profile										
D-dimers (100-560 ng/mL)	N/A	N/A	Raised (2727 ng/mL)	Raised (1207 ng/mL)	N/A	N/A	N/A	N/A	raised (894 ng/mL)	N/A
Fibrinogen (1.99-4.09 g/L)	N/A	N/A	Raised (7.48 g/L)	Raised (597 g/L)	N/A	N/A	N/A	N/A	Raised (18.61 g/L)	Raised (5.24 g/L)
Biochemistry										
LDH (125-243 U/L)	N/A	N/A	Raised (360 U/L)	Raised (301 U/L)	N/A	N/A	N/A	N/A	N/A	N/A
Albumin (35-54 g/L)	Decreased (20 g/L)	N/A	N/A	N/A	Decreased (28 g/L)	Decreased (26 g/L)	Decreased (30 g/L)	Decreased (21 g/L)	Decreased (22 g/L)	Decreased (27 g/L)
Cardiac markers										
Troponin (0-15 ng/L)	Raised (60 ng/L)	N/A	Raised (84 ng/L)	Raised 112 ng/L)	N/A	N/A	N/A	Raised (29 ng/L)	normal	N/A
NT-proBNP (<300 pg/mL)	N/A	N/A	Raised (9477 pg/mL)	Raised (8718 pg/mL)	N/A	N/A	N/A	Raised (8000 pg/mL)	Raised (3131 pg/mL)	N/A
Blood profile										
White cell count (4-15.5 x 10^9^)	Raised	Raised	Raised	Raised	Raised	Raised	Raised	Raised	normal	Slightly raised
Lymphopenia	N/A	N/A	yes	yes	N/A	N/A	N/A	yes	N/A	N/A
Hemoglobin (111-147 g/L)	Decreased	N/A	Normal	N/A	Decreased	Decreased	Decreased	N/A	N/A	Slightly decreased
Platelets (200-450 x 10^9^)	Decreased	N/A	Normal	Normal	Normal	Normal	Normal	Normal	Decreased	Decreased
Chest X-ray	Cardiomegaly	N/A	N/A	N/A	Faint opacity in the left mid-lung zone	Right upper and middle lobe infiltrates	Normal	Cardiomegaly	Cardiomegaly	Pulmonary infiltrates at the right base with accentuated bronchovascular markings in bilateral perihilar and para-cardiac regions

Treatment strategies focused on the management of KD-like features as well as the symptomatic treatment of COVID-19 (Table 6). For KD-like illness, 9 (90%) out of 10 cases received intravenous immunoglobulin (IVIG) therapy, whereas 7 (70%) of them received acetylsalicylic acid as well. The symptomatic treatment for COVID-19 included antibiotics (60%), oxygen (40%), steroids (30%), tocilizumab (20%), anticoagulation, plasma therapy, and antivirals (10%).

**Table 6:** Treatment strategies IVIG, intravenous immunoglobulin

Treatment	Patient 1	Patient 2	Patient 3	Patient 4	Patient 5	Patient 6	Patient 7	Patient 8	Patient 9	Patient 10
IVIG therapy	Yes	Yes	N/a	Yes	Yes	Yes	Yes	Yes	Yes	Yes
Acetylsalicylic acid	Yes	No	N/a	No	Yes	Yes	Yes	Yes	Yes	Yes
Oxygen	Yes	No	N/a	No	No	Yes	No	Yes	No	Yes
Steroids	Yes	No	N/a	Yes	No	No	No	Yes	No	No
Antibiotics	No	No	N/a	Yes	No	Yes	Yes	Yes	Yes	Yes
Tocilizumab	No	No	N/a	Yes	No	Yes	No	No	No	No
Anticoagulant	No	No	N/a	Yes	No	No	No	No	No	No
Antiviral therapy	No	No	N/a	Yes	No	No	No	No	No	No
Plasma therapy	No	No	N/a	Yes	No	No	No	No	No	No

## Discussion

KD is an acute and self-limiting vasculitis that is characterized by the inflammation of the medium caliber vessels. It almost exclusively affects children [7]. In the acute phase, patients with KD might develop hemodynamic instability, a condition known as KD shock syndrome (KDSS) [8]. KD is the chief cause of acquired heart disease among children in developed countries resulting in detrimental complications such as coronary artery ectasias, cardiac inflammation, and aneurysms in 15-25% of patients if left untreated. KD may also cause myocarditis, pericarditis, and abnormal heart rhythms [1].

There are published studies regarding the presence of KD-like features in pediatric COVID-19 infection. The pathophysiology of KD and COVID-19 is similar, i.e., exaggerated inflammatory response (cytokine storm) leading to vascular endothelial damage and immune-mediated tissue injury. This may account for the overlapping of their symptoms [9]. COVID-19 patients also demonstrate KD-like coronary changes, as seen in two (20%) of the cases included in our study who developed coronary artery dilation. None of the patients developed a coronary aneurysm. Pericardial effusion was seen in four (40%) patients and myocarditis was seen in three (30%) patients. In another study, 19 (90%) out of 21 children and adolescents (median age: 7.9 years) who were admitted with features of KD over 15 days showed evidence of recent SARS-CoV-2 infection. Also, 12 (57%) presented with KD shock syndrome and 16 (76%) with myocarditis, with 17 (81%) requiring intensive care support. All of them had significant gastrointestinal symptoms during the early stage of illness and increased inflammatory markers. Each one of them had a favorable clinical outcome. Moderate coronary artery dilations were detected in five (24%) of the patients [10].

Typical KD has certain differences from the pattern seen in COVID-19 patients. Although KD can affect the pediatric population of any race, those with Asian origin are more prone to it [1]. This may suggest that in COVID-19 infection, KD-like symptoms can occur irrespective of the racial predisposition. Moreover, the cases included in our study were reported between April and July. This pattern is also different from the usual winter predisposition of KD, as indicated by the three KD epidemics recorded in Japan in 1979, 1982, and 1986, with the highest incidence seen in January [11]. In a study, the Kawasaki-COVID-19 cohort differed from a comparator group of ‘classical’ KD by older age at onset (10 vs 2 years; p<0.0001), lower platelet count (188 vs. 383 G/L; p<0.0001), a higher rate of myocarditis (7/16 vs. 3/220; p=0.0001), and resistance to first IVIG treatment (10/16 vs. 45/220; p=0.004) [12].

The cause of KD remains unknown, although half a century has passed since KD's first case was reported [7]. The most accepted hypothesis supports an aberrant response of the immune system to one or more unidentified pathogens in genetically predisposed patients [13]. It is worth mentioning here that KD lacks a definitive association with any single etiological agent, and multiple infectious triggers such as rhinovirus, parainfluenza virus, respiratory syncytial virus, adenovirus [14], human coronavirus (HCoV-229E) [15], and new haven coronavirus (HCoV-NH) [16] have been linked to it. However, some studies have disproved the connection of human coronavirus with KD [17]. Hence, it is yet to be ascertained whether the detection of SARS-CoV-2 in children with associated KD symptoms should be treated under a diagnosis of KD, or a new separate diagnosis of COVID-19 infection is required. There is a need to differentiate an incidental SARS-CoV-2 infection with concomitant KD from KD triggered by SARS-CoV-2.

Our study included only 10 cases, which limits its reliability due to the small sample size. However, KD criteria were met in all of them (100%), with a complete presentation in 5 (50%). Fever was present in all patients. The conjunctival and oral cavity changes, including rash, were the most common features. Seven (70%) children required admission to a critical care unit, but no mortality occurred. This article helps understand and deal with KD-like manifestation of pediatric COVID-19 infection, especially in critical care settings, and its possible complications. It will help in timely and appropriate decision-making regarding treatment and management of such cases. More studies on a large scale are required to prove any association between pediatric COVID-19 infection and KD. Yet, to be on the safe side, it is advised that care should be taken when reintegrating these pediatric patients in the society.

## Conclusions

The clinical spectrum of COVID-19 symptoms in children is yet to be defined, and no guidelines have been established regarding screening of COVID-positive pediatric patients for KD. Lack of timely diagnosis and management of concurrent KD can prove detrimental. Hence, the children diagnosed with KD should be tested for SARS-CoV-2, the pathogen for COVID-19, to differentiate the etiology from other infectious triggering agents and avoid missing underlying COVID-19.
